# Potential Therapeutic Treatments for Doxorubicin-Induced Cardiomyopathy

**DOI:** 10.7759/cureus.21154

**Published:** 2022-01-12

**Authors:** Shadman Kabir, Nimisha Lingappa, Harvey Mayrovitz

**Affiliations:** 1 Osteopathic Medicine, Nova Southeastern University Dr. Kiran C. Patel College of Osteopathic Medicine, Davie, USA; 2 Medical Education, Nova Southeastern University Dr. Kiran C. Patel College of Allopathic Medicine, Davie, USA

**Keywords:** left ventricular ejection fraction, reactive oxygen species, echocardiograph, cardiotoxicity, cardioprotective, cardiomyopathy, doxorubicin

## Abstract

Doxorubicin (DOX) is an anthracycline antibiotic used to treat many cancers, including breast cancer, leukemia, and Hodgkin’s lymphoma. Positive aspects of DOX use are limited by the cardiomyopathy that it may cause. For this reason, it is crucial to uncover effective treatments against DOX-induced cardiomyopathy (DIC). Oxidative stress plays a pivotal role in DIC, and this understanding has helped guide potential treatments for DIC. The purpose of this study was to review and describe current and emerging treatments for DIC and their potential cardioprotective effects against DIC. The goals were to: (1) provide a single-source report to aid clinicians in exploring different treatment plans that are personalized for their patients and (2) stimulate researchers to consider evaluating promising and emerging treatment modalities. Evolving understanding of DIC pathophysiology remains fundamental in elucidating the course for future medical therapies. The main conclusion of this study was that the use of existing pharmaceutical agents might represent a possible approach towards mitigating DIC with more extensive clinical data. A limitation of all reviewed studies was that none included an experimental model in which DOX was used to treat animals with preexisting cancer. This limitation fails to identify the impacts that cancer may play in DIC pathophysiology and the potential mitigating effects of DIC treatments. Future research should consider this limitation with appropriately revised protocols.

## Introduction and background

**Figure 1:**
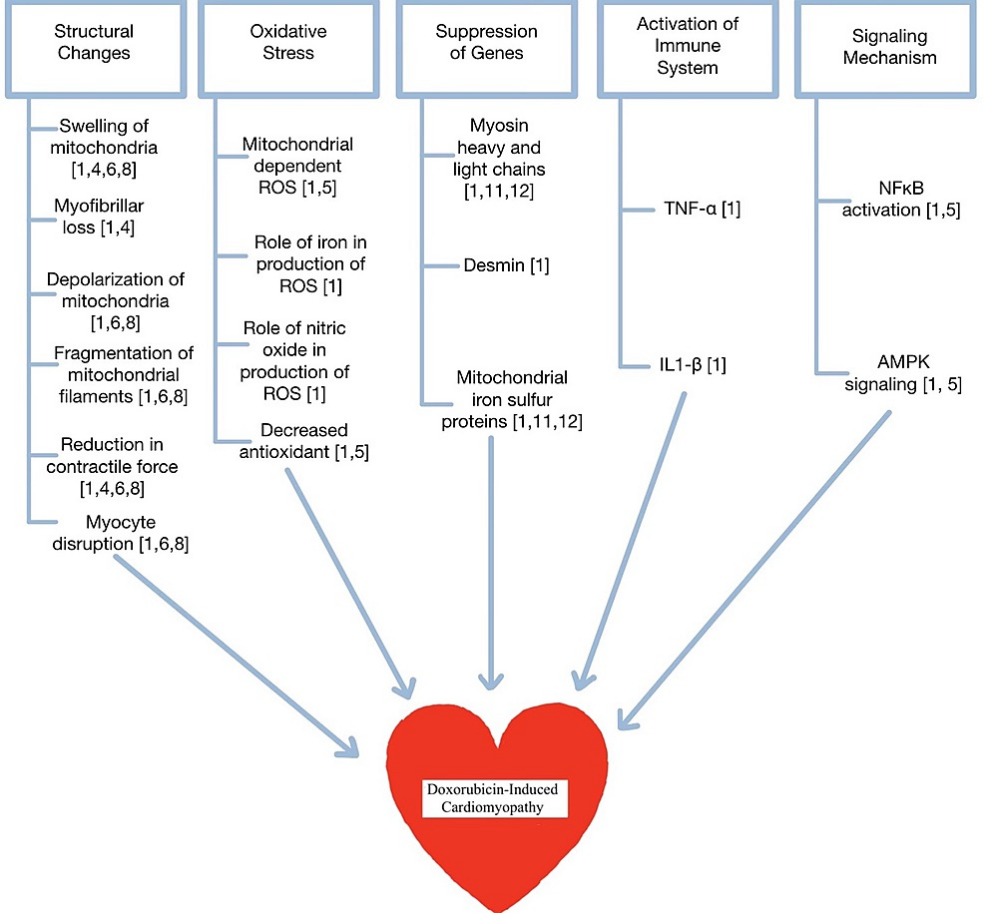
Doxorubicin-induced cardiomyopathy mechanisms Changes seen in DIC such as structural changes, increased oxidative stress, gene suppression, immune system activation, and the upregulation of signaling mechanisms all contribute to the pathogenesis of DIC. ROS: reactive oxygen species, TNF-a: tumor necrosis factor alpha, IL1-b: interleukin 1 beta, NFkB: nuclear factor kappa-light-chain-enhancer of activated B cells, AMPK signaling: AMP-activated protein kinase [1,5-12]. Image credits: Nimisha Lingappa and Shadman Kabir

Doxorubicin (DOX), under the brand name Adriamycin, is an anthracycline antibiotic used as a chemotherapeutic agent to treat many types of cancers, including solid and hematogenous cancers [1]. DOX is administered via a central line or peripheral venous catheter through a slow infusion to avoid the risk of adverse reactions. DOX’s primary mechanism of action involves intercalating DNA base pairs resulting in DNA strand breakage and prevention of DNA and RNA synthesis. DOX can cause further DNA damage and stimulate cellular apoptosis by inhibiting the enzyme topoisomerase II. This enzyme aids in DNA replication and transcription by altering DNA supercoiling and unlinking the double-stranded DNA segments [2]. In addition, DOX in combination with iron can lead to reactive oxygen species (ROS) mediated DNA damage [3]. Although DOX is an effective cancer treating agent against many types, including breast, ovary, bladder, myeloblastic leukemia, Hodgkin lymphoma, and small cell lung cancer, it has the significant cardiac side effect of cardiomyopathy [3]. As a consequence of DOX-induced cardiomyopathy (DIC), the clinical advantages of DOX are drastically reduced. After a cumulative dosage of 400-700 mg/m^2^ in adults and 300 mg/m^2^ in children, DOX creates a toxicity level that intrinsic antioxidant mechanisms cannot eliminate [1]. The primary way DIC occurs is through increased oxidative stress. Multiple mechanisms trigger this oxidative stress, and these will be discussed, subsequently along with the different drugs and therapies that target them. In addition to the oxidative stress, DIC leads to the alteration of subcellular organelles, including mitochondrial swelling, increased lysosomes, myofibrillar loss, and nucleus abnormalities. These organelle changes eventually lead to reduced contractile force and myocardial dysfunction. The various mechanisms of DIC damage can be seen in Figure 1, displaying structural changes associated with DIC. These changes have been compared to dilated cardiomyopathy due to features such as four chamber dilation and reduced ventricular ejection fraction [4].

As shown in Figure 1, oxidative stress is a key issue that results from DIC. Oxidative stress occurs when the cell's antioxidant capacity is unable to match the production of ROS and reactive nitrogen species [5]. When the DOX cumulative dosage surpasses 400-700 mg/m^2^, the DOX-induced oxidative stress exceeds normal intrinsic antioxidant mechanisms [1]. The oxidative stress associated with DIC occurs due to mitochondrial disruptions, increased intracellular iron, increased nitric oxide levels, and decreased antioxidants. In addition to the oxidative stress-related effects, there are numerous other effects. Some such effects include dysregulated lipid metabolism [6], decreased ATP production [7], mitochondrial swelling [6], mitochondrial membrane disruptions [8], and increased development of ROS [9]. All of these changes can lead to the mitochondrial dysfunction and apoptosis of cardiomyocytes seen in DIC. Further, the immune system releases a range of cytokines during DOX treatment that results in a change in immune cells, which impacts cardiac function [1]. DIC has been linked to increased expression of toll-like receptors 2 and 4, principally involved in cardiac pathogenesis, which, when activated, leads to the production of nuclear factor kappa B, which promotes apoptosis [10]. The genes of vital contractile proteins such as troponin I, troponin C, alpha-actin, myosin heavy and light chains, and desmin are all downregulated in DIC [11-12].

Early manifestations of DIC include chest pain and/or palpitations commonly due to sinus tachycardia, paroxysmal non-sustained supraventricular tachycardia, or premature atrial and ventricular beats [4]. Electrocardiogram changes such as tall, broad P-waves with a dart-and-dome configuration in lead V1 indicative of bi-atrial enlargement and a QRS complex revealing left bundle branch block with left axis deviation can also be seen in DIC [13]. However, with the termination of DOX, acute changes unrelated to dosage such as myopericarditis are reversible, and sinus rhythm can be restored [13]. A higher incidence of DIC can be seen in patients administered with a higher total dose, children under four years, adults of advanced age, especially females, and those with pre-existing cardiovascular disorders [14]. Further studies have shown that approximately 9% of DIC is dose-dependent in adult patients, and most symptoms are observed within the first year of chemotherapy [10]. However, earlier detection of cardiac function changes leads to improvement in the patient’s condition over time.

Dexrazoxane, an iron chelator, may mitigate such DNA damage by inhibiting DOX binding to iron. This drug is used as a cardioprotective treatment to help mitigate DIC [3,15]. However, iron chelation alone might not be sufficient to protect against anthracycline-induced cardiomyopathy and could potentially induce myelosuppression [3,16]. Thus, at present, the prophylactic and treatment options for DIC are quite limited.

Before the employment of DOX, heart function tests, including electrocardiogram or echocardiogram, are conducted to determine baseline functioning. A complete blood count is done before treatment to monitor one of the known side effects of DOX, bone marrow suppression [3]. In addition, some physicians conduct exercise stress tests to determine if the left ventricular ejection fraction (LVEF) is adequate for toleration of treatment with DOX [17]. In these preliminary tests, if a patient has an abnormal heart rate, decreased LVEF, or low blood cell counts, DOX is contraindicated. If DOX is administered due to proven patient stability, routine tests are done throughout drug therapy. Some limitations exist in detecting reversible cardiac damage via routine physical and electrocardiogram, so a radionuclide angiography can be conducted [17]. Serial radionuclide angiography provides more accurate information about LVEF changes throughout DOX treatment to monitor for possible cardiotoxicity. New routine tests have also been added to measure the left ventricular filling rate during diastole via the same standard radionuclide evaluation [17]. If a reduced LVEF is discovered through routine testing via echocardiography or radionuclide angiography, DOX is discontinued immediately [3]. Therefore, to detect DIC as early as possible, a careful pretreatment assessment, including electrocardiogram, chest radiograph, echocardiogram, radionuclide ventriculography, or nuclear imaging [4], is vital.

## Review

The purpose of the present review is to systematically evaluate recent findings regarding emerging pharmaceutical agents that might serve as potential novel treatments for DIC. The goal is to produce a single source-focused document to help clinicians consider and investigate promising treatment options not yet fully vetted.

Methods

The following five databases were searched for peer-reviewed articles written in English and published from 2011-2021, inclusive: PubMed, Web of Science, Embase, CINAHL, and Biomedical Reference Collection: Comprehensive. These databases were searched using the keywords “Doxorubicin” and “Cardiomyopathy” in the title and “Protect*” in the abstract. The asterisk (*) served as a wild card to include possible variations of the word protect, such as protection, protected, or protecting. After removing duplicates from this search, a total of 130 papers met the keyword search criteria. These 130 articles were filtered according to the following inclusion/exclusion criteria. Papers were included if they contained studies on humans or experimental animals and incorporated quantitative assessments of cardiac function as part of the research or clinical study. Gene therapy and in vitro studies were excluded. After eliminating studies according to these parameters and adding additional papers obtained from references within these articles, 48 papers remained for final analysis.

Left ventricular dysfunction and mitochondrial function as a pharmaceutical target

DIC often causes left ventricular remodeling that leads to a reduced LVEF [4]. A DOX-related surge in cellular oxidative stress causes increased cell death that contributes to this pathophysiological process. The mitochondria play a vital role in alleviating the effects of this oxidative stress, and DOX-related mitochondrial dysfunction reduces this mitigation leading to cell death [1]. Thus, multiple pharmacological agents target this DOX-related effect on mitochondria.

An in vivo approach being evaluated for its protective effects against DIC in a mouse model is cannabidiol, a non-psychotropic constituent of marijuana. Cannabidiol is reported to attenuate the effects of mitochondrial dysfunction induced by DOX due to its potent antioxidant and anti-inflammatory effects [18]. The restorative ability of cannabidiol is indicated by the combination of improved complex I activity and reduced oxidative stress in myocytes [18]. With the use of cannabidiol, DOX-induced increases in creatine kinase, a marker of cardiac injury, were decreased. In addition, cardiac dysfunction evaluated by LVEF was greatly attenuated, and cardiomyocyte function was retained.

A possible correlation between mitochondrial effects and impact on cardiac function has been examined using carvedilol, a cardioselective beta-blocker with additional alpha-1 blocking activity in a mouse model. In the in vivo portion of this study, mitochondrial integrity was measured by analyzing the cytochrome C to the beta-tubulin ratio [19], with cytochrome C used as an indicator of mitochondrial damage induced by DOX. Mice in a control group were found to have the lowest cytosolic cytochrome C to the beta-tubulin ratio of 0.1 compared to the DOX-only treatment group with a ratio of 1.4. However, the combined treatment of carvedilol and DOX was found to have a ratio of 0.3, suggesting a mitigating effect of the treatment. The significant reduction of cytochrome C levels by carvedilol reinforces its ability to improve mitochondrial dysfunction associated with DIC. A similar trend of improvement was seen in LVEF. The control group had an LVEF of 60%, and the carvedilol and DOX combined therapy group had an LVEF of 58%. These values contrast with the DOX-only treatment group with an LVEF of 41% [19]. The values of the combined treatment group of DOX and carvedilol suggest preservation of mitochondrial function and decreased cardiac myocyte death.

Another study also investigated the cardioprotective effects of carvedilol in combination with liposomal resveratrol against DIC in rats [20]. Carvedilol, in combination with resveratrol, a natural polyphenol with anti-inflammatory and anti-apoptotic effects, exerts antioxidant effects by decreasing the generation of ROS and restoring mitochondrial function. With the administration of DOX alone, increases in creatine kinase activity by 40.6% and troponin-1 levels by 60.6% were seen [20]. On the other hand, with combined treatment of carvedilol and resveratrol, decreases in creatine kinase activity by 35% and troponin-1 levels by 81.35% were measured [20]. The changes in this study help to provide further evidence of the potential therapeutic events of carvedilol against DIC by decreasing serum levels of cardiac damage biomarkers after treatment with DOX.

In contrast to carvedilol in mouse models, a prospective, randomized, double-blind study of patients was conducted to determine if prophylactic use of carvedilol can prevent DIC and if its effects are dose-dependent [21]. Echocardiogram data showed LVEF of patients treated with DOX and carvedilol at dosages of 6.25 mg, 12.5 mg, and 25 mg had no significant improvement in LVEF at six months when compared to patients treated with DOX and a placebo in the same time frame [21]. This study fails to provide conclusive clinical data on carvedilol’s ability to mitigate cardiotoxicity in DIC and suggests the need for further studies.

A potential mitigating approach using hydrogen sulfide was evaluated in a rat model. Mitochondrial function was evaluated by measuring the levels of malondialdehyde, superoxide dismutase, glutathione peroxidase, and ROS [22]. In vivo data showed hydrogen sulfide was able to increase superoxide dismutase and glutathione peroxidase and decrease malondialdehyde, thus reducing overall cellular oxidative stress. These decreased stress levels allowed for less myocyte apoptosis and ultimately a lessened decline in LVEF. 

Co-enzyme Q10 is an essential part of the mitochondrial electron transport chain and is a powerful antioxidant in many nutritional supplements given alongside cancer treatment. An in-vivo rat model was conducted with oral Q10 use as a potential preventative method in DIC [23]. With the administration of DOX only, hematoxylin and eosin (H&E) staining revealed an accumulation of abnormal collagen within cardiomyocytes. This collagen increase was not observed with the combined administration of Q10 with DOX, which indicates a potential amelioration of the cardiac damages induced by DOX and an improvement in cardiac health.

All four of the previously discussed agents, cannabidiol, carvedilol, hydrogen sulfide, and Q10, show some promise to decrease the stress placed on various aspects of mitochondria that are affected in DIC. By limiting the negative effects, these potential therapies can help maintain an adequate and sustainable LVEF. Further studies are needed to bring these to the next level of acceptance.

Emerging procedures with potential cardioprotective effects

A novel recent study investigated repurposing extracorporeal shock wave (ESW) therapy to treat DIC [24]. Using an in vivo mouse model of DIC, the data reported that prophylactic ESW treatment, in the form of 1,000 short high energy pulses, improved left ventricular function. ESW was delivered to the animals one hour before DOX treatment and then three times a week for the next two weeks. Echocardiographic measurements of LVEF showed that the animals administered DOX but treated with ESW had an LVEF of 70% compared with a healthy control group that had an LVEF of 75%. These values contrast to an LVEF of 35% in animals given DOX without the ESW treatment. Additional in vitro data from this study provided evidence that the ESW therapy was associated with upregulating survivin, an inhibitor protein that regulates cellular apoptosis and tumor progression [24]. The anti-apoptotic activity of survivin in the myocardium was confirmed by measuring Bcl2, an anti-apoptotic protein, in the presence or absence of YM155, a survivin inhibitor.

Pharmaceutical agents with potential cardioprotective effects

A potential alternative approach to mitigating DIC is through the inhibition of aldose reductase, an enzyme with a critical role in oxidative stress-induced inflammatory signals [25]. In a recent study, the drug fidarestat, an aldose reductase inhibitor in phase 3 clinical trials for the treatment of diabetic nephropathy, was proposed to be effective at ameliorating DIC. The study measured troponin I levels and used echocardiography to monitor the effects of the drug on DIC. The in vivo data showed that LVEF was significantly improved from 39% in the DOX-only treatment group to 48% in the DOX plus fidarestat group [25]. Data also showed fidarestat was able to significantly reduce serum troponin I level from 3 ng/mL in the DOX-only group to 1.3 ng/mL in the DOX plus fidarestat group. The trend in troponin levels indicates the reduction of cardiomyocyte death and maintenance of function. However, the study was unable to provide statistically significant data (p=0.06) [25] on troponin I levels and ultimately determine its impact on cardiotoxicity. 

Statins and angiotensin receptor blockers have cardioprotective effects and are commonly used to reduce blood pressure and improve left ventricular function in patients with heart failure [26]. An in vivo study using a rat model of DIC highlights cardioprotective effects of the combination of rosuvastatin, a commonly used statin, and candesartan, an angiotensin receptor blocker; since both have different mechanisms of action [26]. The induction of cardiomyopathy in this experimental model occurs from a combination of DOX and trastuzumab, a monoclonal antibody used to treat breast and stomach cancer that has been shown to worsen the cardiotoxicity associated with DIC [27]. Masson’s trichrome stain was used to evaluate the degree of fibrosis and showed that the rate of cardiac fibrosis was favorably impacted by the combined treatment of rosuvastatin and candesartan against the resultant DIC [26]. Fractional shortening rate and longitudinal strain were used to track ventricular function. With the use of 2-D M-Mode echocardiography, fractional shortening rate is an easy and reliable measure that represents the contractile function of the left ventricle [28]. Using 2-D speckle tracking software, the longitudinal strain rate is a useful echocardiographic technique to evaluate left ventricular dysfunction by recording data on systolic and diastolic functioning [29]. The longitudinal strain has been observed to detect left ventricular dysfunction earlier in cancer patients than changes in LVEF [30]. The fractional shortening and longitudinal strain rates showed there was an improvement in cardiac function by the combined treatment of rosuvastatin and candesartan against DIC. These findings, along with positive effects on high sensitivity C-reactive protein and left ventricular dP/dt, indicate the potential of such treatment in DIC [26].

Under normal conditions, alpha-1 adrenergic receptors represent about 10% of cardiac adrenoreceptors but are seen in increased numbers in heart failure and may serve to help preserve declining function [31]. The mitigating effects of compound A61603 (A6), a potent and selective imidazoline agonist of alpha-1A, were evaluated in a rat model of DIC [32]. The method used to induce cardiomyopathy was to deliver a single dose of 250 ml saline intraperitoneally as a drug vehicle with a DOX dose specific to rat gender and weight. Echocardiography was used to detect changes in cardiac function and hemodynamics, and creatine kinase activity was measured to determine the rate of myocyte necrosis. In vivo echocardiographic data revealed similarities in pre-DOX and post-DOX treatment with A6 mice with fractional shortening rates of 58% and 61%, respectively [32]. In contrast, post-DOX untreated mice were seen to have a fractional shortening rate of 49%. These numbers support the idea that A6 can alleviate the myocardial dysfunction caused by DIC and even enhance myocardial functioning before DIC. The effects of treatment with A6 in DIC were also reflected in cardiac output measurements and preservation of hemodynamics. Cardiac output values were calculated using stroke volume and heart rate from data collected by the echocardiogram. Mice receiving only the DOX vehicle were seen to have a decrease in heart rate by 55% when compared to post-DOX mice treated with A6 with a 23% decrease in heart rate. A similar trend is also seen with stroke volume with a 50% decrease in untreated mice versus a 33% decrease in mice treated with A6. Together, these values created calculated cardiac outputs for pre-DOX, DOX plus A6, and untreated post-DOX of 22%, 13%, and 6%, respectively. An increase in the cardiac output suggests maintenance of cardiac function with the use of A6 as an alpha-1A agonist. 

Another agent, Cilostazol, a phosphodiesterase III (PDE-3) inhibitor, has been reported to have in vivo therapeutic effects in acute decompensated dilated cardiomyopathy comparable to DIC [33]. Evaluation of the cardioprotective effects of Cilostazol has been undertaken in a rat model of DIC [34]. In this study, rats were given DOX only or DOX combined with cilostazol, and echocardiography was conducted to assess fractional shortening rates as an index of cardiac function. Fractional shortening rates were calculated to be similar in control animals without DOX treatment and those with combined DOX and cilostazol treatment with fractional shortening rates of 68% and 58%, respectively [34]. In contrast, the DOX-only treatment group was calculated to have a fractional shortening rate of 49%. Another indicator of cilostazol’s potential benefit was the alteration on brain natriuretic peptide, which is commonly secreted by myocytes in response to cardiac overload. Brain natriuretic peptide was significantly higher in the DOX-only treatment group at 340 pg/mL compared to the combined DOX and cilostazol treatment group at 170 pg/mL. This lower level of brain natriuretic peptide suggests cilostazol plays a role in protecting against left ventricular dysfunction seen in DIC.

Thiamine, also known as vitamin B1, is a water-soluble vitamin that plays importance in numerous enzymatic reactions of cellular metabolism [35]. A deficiency in thiamine leads to increased oxygen consumption, myocardial dysfunction, and eventual cardiomyopathy. Researchers aimed to study the effects of a seven-day treatment plan with intraperitoneal thiamine injections to provide cardioprotection against DIC in a rat model. Rat hearts studied via echocardiography revealed a single injection of DOX decreased LVEF from 50.9% to 38.2%. On the other hand, the combination of a single DOX injection and seven-day thiamine treatment only decreased LVEF from 51% to 46.7% [35]. Treatment with thiamine limited the drop in LVEF and maintained more cardiac function. In addition, after a single DOX injection, free radicals of oxygen increased by 89% compared to only 19.4% in the co-administration of DOX and thiamine [35]. Lower levels of free radicals limit oxidative stress placed on the heart in DIC.

Dietary supplements with potential cardioprotective effects

A Mediterranean diet has been reported to have mitigating effects on cardiovascular diseases, including hypertension and cardiac hypertrophy, and was evaluated for its efficacy in mitigating DIC in a rat model [36]. The cardioprotective effects of extra virgin olive oil, a monounsaturated fat commonly found in Mediterranean diets, was assessed in a five-group in vivo study, including normal control, DIC control, and three groups that, in addition to a standard diet, received olive oil at different concentrations (2.5%, 5.0%, and 10.0%). The main finding reported from this study was that supplementation of the diet with 10% olive oil served to ameliorate DIC as judged by lessened values of cardiac markers [36]. Most notably, this group had a troponin T level of 0.39 ± 0.03 ng/mL compared to 1.80 ± 0.14 ng/ml in the DIC group with the standard diet and without olive oil [36]. There was also a positive effect on lipid peroxidation and antioxidant enzyme levels, including malondialdehyde and superoxide dismutase for the 10% olive oil group. The lower malondialdehyde levels paired with higher levels of superoxide dismutase allowed for cells to ameliorate the oxidative stress associated with DIC, similar to the mechanism of action of hydrogen sulfide previously mentioned [22].

Other researchers have also reported the positive benefits of components of olive oil in relation to DIC. Oleuropein, a natural phenolic antioxidant found in olive oil and responsible for the bitter taste and strong aroma, was studied as one such potential cardioprotective agent [37]. Six treatment groups were created with DOX administration and different oleuropein treatment plans ranging from either 100 mg or 200 mg doses in a matter of three or five days [37]. Blood samples were taken from the rats 72 hours after DOX administration, and creatine kinase levels were measured. Creatine kinase in the DOX-only treatment group was around 9,500 IU/L, but all treatment groups with a combination of DOX and oleuropein were significantly lower, ranging from 3,500 IU/L to 4,000 IU/L [37]. These decreased values indicate a reduction in oxidative stress and ROS accumulation leading to a reduction in myocardial tissue death induced by DIC. Current data suggest that olive oil has a high antioxidant capacity in a concentration-dependent manner to improve cardiotoxicity in DIC.

Dietary polyphenols, specifically flavonoids, known to have some cardioprotective effects due to their antioxidant and anti-inflammatory properties, have been evaluated in the context of DIC treatment [38]. The use of a mix of flavonoids extracted from the fruit of the Bergamot plant has been assessed as a way to reduce oxidative stress linked to DIC in a rat model [38]. The full details of this study are unclear since only hemodynamic, histological, and biochemical analyses were reported. Echocardiography was used to monitor left ventricular function, while histological analysis was used to evaluate myocyte apoptosis. In addition, 8-OHdG, a marker of DNA oxidative damage, was measured via staining of cardiomyocytes to evaluate the antioxidant capacity of flavonoids extracted from Bergamot. The preliminary data suggest that flavonoids from Bergamot improved left ventricular function, reduced myocyte apoptosis, and prevented oxidative damage [38]. The author states that flavonoids of Bergamot significantly prevent 8-OHdG accumulation in cardiac tissue, but with no values provided by the study, the efficacy of flavonoids from Bergamot to mitigate DIC is unable to be determined. However, in 2018 an update to the study was conducted. The DOX plus Bergamot flavonoids group and control group were found to have limited ROS accumulation shown by 8-OHdG staining of 0.167% and 0.12%, respectively [39]. However, in the DOX-only treatment group, there was a significant increase to 1.042% in 8-OHdG positively stained cardiomyocytes indicating increased ROS accumulation [39]. This study also focused on the effect of Bergamot in promoting endogenous cardiac stem and progenitor cells. The activation of these cells helped support new cardiomyocyte formation to maintain an effective barrier against ROS created in DIC. The reestablishment of protective cells is evident by the combined treatment of DOX and Bergamot flavonoid with 0.06% stem cells remaining of the total cell population compared to the DOX only group with 0.025% cardiac stem and progenitor cells available [39].

The demand for natural remedies has recently increased. An in vivo study used a rat model of DIC to evaluate the efficacy of hesperidin, a flavanone glycoside found in citrus fruits, and vitamin E, a lipid-soluble vitamin with extensive free radical scavenging functions, as natural remedies to treat DIC [40]. To track cardiomyocyte damage, the study measured creatine kinase, which is a sensitive biomarker released from cardiac cells after direct tissue injury or change in permeability [41]. To track apoptosis changes in caspase 3, a protease that is released from cells during programmed cell death was also tracked [42]. Reduction in levels of either creatine kinase or caspase 3 may indicate the experimental treatment has mitigating effects. The control group had a creatine kinase level of 1300 U/L while the DOX plus hesperidin treatment group and DOX plus vitamin E treatment group had creatine kinase values of 1400 U/L each [40]. These values contrast with the DOX-only treatment group that had a creatine kinase level of 2000 U/L. Furthermore, the control group had a cardiac caspase 3 level of 100%, while the DOX plus hesperidin and DOX plus vitamin E groups had cardiac caspase 3 levels of 146% and 149%, respectively [40]. Similarly, these values differ from the DOX-only treatment group with a cardiac caspase 3 levels of 220% [40]. Overall, the in vivo data suggests that hesperidin and vitamin E can alleviate the cardiotoxicity and myocyte apoptosis associated with DIC. 

Similar to these natural remedies, two different in vivo studies were conducted to evaluate the cardioprotective effects of pycnogenol, an aqueous extract from pine bark with bioactive flavonoids and methanolic extract from the Ixora coccinea plant [43,44]. The framework of the study involving pycnogenol had five treatment groups: a control group, a DOX only group, a pycnogenol only group, a prophylactic pycnogenol plus DOX group, and a post-DIC pycnogenol plus DOX group [43]. Pycnogenol mitigated the cardiotoxicity caused by DIC, evidenced by significantly lower levels of malondialdehyde which is an indicator of oxidative stress. In both the prophylactic treatment group and post-DIC treatment group, malondialdehyde was 12.57 nmol/ml and 9.51 nmol/ml, respectively [43]. In contrast, the DOX-only treatment group had a malondialdehyde level of 16.27 nmol/ml [43]. This same cardioprotective effect can be seen with increased levels of glutathione, an antioxidant preventing damage by ROS, in the prophylactic and post-DIC treatment groups when compared to the DOX-only treatment group. When comparing the prophylactic pycnogenol group to the post-DIC pycnogenol group, the difference in troponin T levels suggests that treating DIC with pycnogenol has a greater effect once the DIC has already occurred. The troponin T level in the prophylactic treatment group was 45.19 pg/ml, and in the post-DIC treatment, the group was 28.55 pg/ml as opposed to the elevated level of 60.68 pg/ml in the DOX only group [43].

Ixora coccinea (Ixora), commonly known as jungle geranium, is a flowering shrub used in the tropics for nausea, dysentery, dermatological conditions, and even as an antioxidant. Its use to mitigate the effects of DOX treatment was evaluated in a study consisting of four groups of rats; a control group, a DOX only group, a prophylactic Ixora (200mg/kg) plus DOX group, and a prophylactic Ixora (400mg/kg) plus DOX group [44]. In vivo data suggested that prophylactic Ixora use caused normalization of the usually prolonged QT intervals associated with DOX treatment and resulted in a decrease in creatine kinase level from 149.7 U/L in the DOX group to 76.5 U/L in the higher dose Ixora treatment group. The pattern seen with creatine kinase levels suggests the ability of Ixora to potentially mitigate DIC.

Another natural substance, chrysin, a flavone found in honey, mushrooms, and bee propolis, possesses antioxidant and anti-inflammatory properties that could potentially mitigate the effects of DIC [45]. Chrysin’s potential cardioprotective effects in a rat model of DIC were evaluated using ECG, serum marker levels, and antioxidant enzyme activity changes. The in vivo ECG data showed a prolonged QTc interval of 295 ms in the DOX-only group, which is similar to what was reported in the Ixora study [44]. However, healthy control and DOX plus chrysin treatment groups had QTc intervals of 200 ms and 210 ms, respectively. These ECG data indicate that chrysin prevented the abnormally lengthened QTc interval associated with DIC. Further, the use of chrysin was associated with a reduction in creatine kinase levels from 200 U/L in the DOX-only group to 150 U/L in the DOX plus chrysin treatment group [45].

Another natural flavone, acacetin, was studied as a potential protective agent against DIC. Acacetin, from the Chinese medicinal herb snow lotus, is known for its numerous pharmacological properties, such as being an anti-inflammatory, anticancer, and cardioprotective agent [46]. It was used in an in vivo mouse model to demonstrate acacetin’s effects on DIC by activating AMP-activated protein kinase (AMPK) and nuclear factor erythroid-2 (Nrf2) molecules causing the upregulation of antioxidant genes [46]. In this study, mice treated with DOX had an LVEF of 48.5%, but when combined with acacetin, LVEF was 59.1%. In addition, ROS production was also less when DOX plus acacetin (148.1% increase) was compared to DOX alone (222.6% increase) in this mouse model. This data indicates acacetin maintains heart functioning, as evidenced by an increased LVEF and decreased ROS accumulation.

Berberine, similar to an alkaloid initially extracted from a Chinese plant to be used as a broad-spectrum antibiotic, was recently studied for its pharmaceutical uses as an antitumor and cardioprotective agent and evaluated as a mitigating agent for DIC in a mouse model [47]. Berberine pretreatment demonstrated significant increases in superoxide dismutase and glutathione activities along with decreased levels of malondialdehyde activity, thereby mitigating increased oxidative stress normally associated with DIC [47]. In addition, ECG data showed berberine decreased the normally prolonged ST-segment and QRS complex durations and QT intervals of DIC. Similar to the acacetin study [46], berberine pretreatment provided some cardioprotective effects by upregulating mitochondrial function [47].

Cardamonin, a chemical isolated from herbs such as Alpinia katsumadai and Ginkgo biloba, has been reported to have antitumor, antioxidative, and anti-inflammatory effects and has been evaluated as a mitigating agent for DOX effects in a mouse model [48]. It significantly decreased cardiomyocyte apoptosis in a dose-dependent manner by increasing levels of nuclear factor erythroid-2. Similar to the berberine study [47], cardamonin significantly increased superoxide dismutase and glutathione activities while reducing both malondialdehyde and ROS levels. In addition, echocardiographic data showed that the addition of cardamonin significantly improved LVEF.

## Conclusions

DIC remains a complex and multilayered complication of DOX cancer treatment which reduces its clinical application. This review analyzed the use of potential treatments against DIC. The findings illustrated various natural, procedure-based, and pharmaceutical agents that help mitigate the associated changes seen in DIC. However, a potential limitation of all of these studies was that none of the mouse and rat models used to mimic the effects of treating human cancers actually had cancer. Thus, it is unclear if cancer could also play a role in the development of DIC or if the mitigating effects reported would be the same in the experimental animals that were actually being treated for cancer. It may be of value in the future to use DOX in experimental animals such as mice or rats that do have cancer. Our evolving understanding of the pathophysiology of DIC remains fundamental in elucidating the course for future medical therapies. Thus, an organized method that utilizes existing pharmaceutical agents may represent a possible approach towards mitigating DIC and restoring clinicians’ confidence in using DOX as a chemotherapeutic agent. The present review has pointed to and summarized some of these emerging possibilities.
